# Optimal Strategy for Colorectal Cancer Patients' Diagnosis Based on Circulating Tumor Cells and Circulating Tumor Endothelial Cells by Subtraction Enrichment and Immunostaining-Fluorescence In Situ Hybridization Combining with CEA and CA19-9

**DOI:** 10.1155/2021/1517488

**Published:** 2021-12-24

**Authors:** Shimu Luo, Yanghang Ou, Tingjin Zheng, Huihui Jiang, Yibo Wu, Jiangman Zhao, Zhishan Zhang

**Affiliations:** ^1^Department of Clinical Laboratory, Quanzhou First Hospital Affiliated to Fujian Medical University, No. 248 East Street, Quanzhou City, Fujian 362000, China; ^2^Shanghai Zhangjiang Institute of Medical Innovation, Shanghai Biotecan Pharmaceuticals Co., Ltd., 180 Zhangheng Road, Shanghai 201204, China

## Abstract

**Background:**

Cancerous embryo antigen (CEA) and carbohydrate antigen 19-9 (CA19-9) are commonly used in clinical practice to assist in diagnosing CRC. However, their sensitivity is very low. This study aims to investigate the clinical significance of circulating tumor cells (CTCs) and circulating tumor endothelial cells (CTECs) compared with CEA and CA19-9 in the auxiliary diagnosis of colorectal cancer (CRC) patients.

**Methods:**

115 pathologically confirmed CRC patients and 20 healthy controls were enrolled in this study. CTCs and CTECs were enriched and identified by subtraction enrichment and immunostaining-fluorescence in situ hybridization (SE-iFISH). A logistic regression was used to establish a model for the receiver-operating characteristic (ROC) curve analysis, and the diagnostic efficacy of CTCs, CTECs, CEA, CA19-9, and their combinations was analyzed.

**Results:**

The CTC (*P* < 0.0001) and CTEC (*P*=0.0009) level was significantly higher in CRC patients than that in healthy controls. For CRC patients, CTC and CTEC level was significantly correlated with tumor stage and lymph node metastasis status, but not with sex, age, tumor location, and degree of differentiation. The positive rate of CTCs, CTECs, CEA, and CA19-9 in CRC patients was 87.8%, 39.1%, 28.7%, and 26.1%, respectively. To distinguish CRC patients from controls, the area under the curve (AUC) of CTC was 0.889, which was much higher than 0.695 of CTEC, 0.696 of CEA, and 0.695 of CA19-9. Establishing ROC curve by logistic regression algorithm, the highest AUC was 0.935, which combined CTCs with CTEC, CEA, and CA19-9.

**Conclusions:**

CTCs combined with CTEC, CEA, and CA19-9 are useful to improve the diagnostic efficiency, which has high clinical significance in the diagnosis of colorectal cancer.

## 1. Introduction

Colorectal cancer (CRC) is the third most common malignant tumor globally [1], and its incidence and mortality rates have been increasing annually in China. Although medical treatments rapidly developed, the CRC patients' prognosis mainly depends on their stage at diagnosis [2]. In clinical practice, CEA and CA19-9 are commonly used to assist in diagnosing CRC. However, the sensitivity of these two tumor markers is relatively low [3]. Although the sensitivity of digital rectal examination, colonoscopy, and 3D reconstruction of colon computed tomography images is high, these techniques have a low acceptance rate among most asymptomatic patients, resulting in misses in accurate diagnosis. Therefore, there is an urgent need to develop an accurate CRC screening strategy based on noninvasive biomarkers, which could be well accepted and widely used in asymptomatic population.

Liquid biopsy has been studied in recent years, which captures tumor traits in the bloodstream and other body fluids [4]. Circulating tumor cell (CTC) is a potential biomarker for the diagnosis and prognosis of patients with malignant tumors, and lots of studies have introduced its clinical significance in various cancers [5–10]. Moreover, the technological means of enrichment and identification are multifarious and had both advantages and disadvantages. For example, immunomagnetic CTC enrichment based on epithelial cell adhesion molecule (EpCAM) had used to be FDA-approved method [11], but it can't find tumor cells that lose EpCAM expression because of epithelial-mesenchymal transition (EMT). CTCs enrichment exploiting physical features dominated by size-based separation approaches, e.g., filtration will lose smaller CTCs [12].

Subtraction enrichment against hematopoietic cell markers such as CD45 leukocyte antigen and immunostaining-fluorescence in situ hybridization (SE-iFISH) is a widely accepted method, which can effectively distinguish chromosome ploidy in the cytoplasm and tumor marker cytokeratins on the cell surface [13, 14]. However, previous studies always defined CD45−/DAPI+/CEP8 > 2 as CTCs [13–15], and the circulating tumor endothelial cells (CTECs) aneuploidy was often identified as CTCs because endothelial markers were not exploited. CTECs are rare in the peripheral blood of healthy individuals, and increased CTECs reflect significant vascular damage and dysfunction [16]. Several studies have demonstrated CTECs were related to tumor angiogenesis [17] and maybe useful to guide antiangiogenic drugs [18]. However, the clinical significance of CTECs in cancer is still poorly understood.

In this study, we adopted an improved SE-iFISH method developed by Cytelligen (San Diego, CA, USA) [19], which used FISH to identify Chr8 aneuploidy and involved CD31 to distinguish CTECs. It could accurately identify CTCs and CTECs. 115 CRC patients and 20 healthy controls were recruited. CTCs and CTECs in peripheral blood were detected using the improved SE-iFISH method, and besides, CEA and CA19-9 were also tested. We analyzed the correlation between CTCs/CTECs and clinical characteristics. Especially, this study provided an optimal strategy for the diagnosis of CRC by comparing the diagnostic significance of CTC alone versus in combination with CTECs, CEA, or/and CA19-9.

## 2. Materials and Methods

### 2.1. Research Subjects

The study enrolled 115 pathologically confirmed patients with CRC at the Quanzhou First Hospital Affiliated to Fujian Medical University from 2018 to 2019 (Table 1). None of them had received any therapeutic procedures, such as surgery, chemoradiotherapy, or targeted therapy, before collecting blood samples. Among them, 69 were males, and 46 were females, with a mean age of 58.2 ± 12.0 years. In addition, 20 healthy individuals at the same hospital during the same period were selected, including 15 males and 5 females, with a mean age of 51.8 ± 6.7 years. The diagnostic criteria and TNM staging of patients with CRC were based on the American Cancer Society's 8th edition cancer staging system. Among the 115 patients with CRC enrolled in this study, 25 had stage I-II cancer, and 90 had stage III-IV cancer.

### 2.2. CTC Enrichment

To prevent epithelial cell contamination, the initial 2 ml of blood was discarded, and 7.5 ml of peripheral blood (PB) was collected from participants in a sodium citrate anticoagulant tube, mixed well. Human Circulating Rare Cell Subtraction Enrichment kit (Cytelligen, San Diego, CA, USA) was used for CTC enrichment according to the manufacturer's protocol. In brief, 7.5 ml PB samples were centrifuged at 800 × *g* for 8 min following the discard of supernatant above the red blood cell. 3 ml hCTC separation matrix was added to the remaining components with intensive mixing following the centrifugation at 450 × *g* for 8 min. Afterward, the white buffy coat was collected and incubated with anti-CD45 monoclonal antibody-conjugated immunomagnetic particles (150 *μ*l) for 20 min at room temperature, and the leukocytes were removed by magnetic separation. The solution without beads was centrifuged at 450 × *g* for 8 min following rinsed twice at room temperature. Finally, the cell pellet completely mixed with cell fixative was used for coated CTC slides and dried at 32°C for 4 h, which would be identified by iFISH.

### 2.3. Immunofluorescence Staining and FISH

The slides were immersed in saline-sodium citrate buffer (2×) for 10 min following dehydration in ethanol for 2 min. Centromere Probe 8 (CEP8) Spectrum Orange (Cytelligen, San Diego, CA, USA) was added to the CTC slides, which were denatured at 76°C for 10 min and hybridized for 4 h at 37°C. Whereafter, the hybridization slides were darkly incubated with AlexaFluor® 594-conjugated anti-CD45 IgG and AlexaFluor® 488-conjugated anti-CD31 IgG for 2 h at room temperature. Finally, DAPI was added to the CTC slides for counting (Cytelligen, San Diego, CA, USA), and CTCs were observed by a fluorescence microscope.

### 2.4. CTC and CTECs Identification

**Figure 1:**
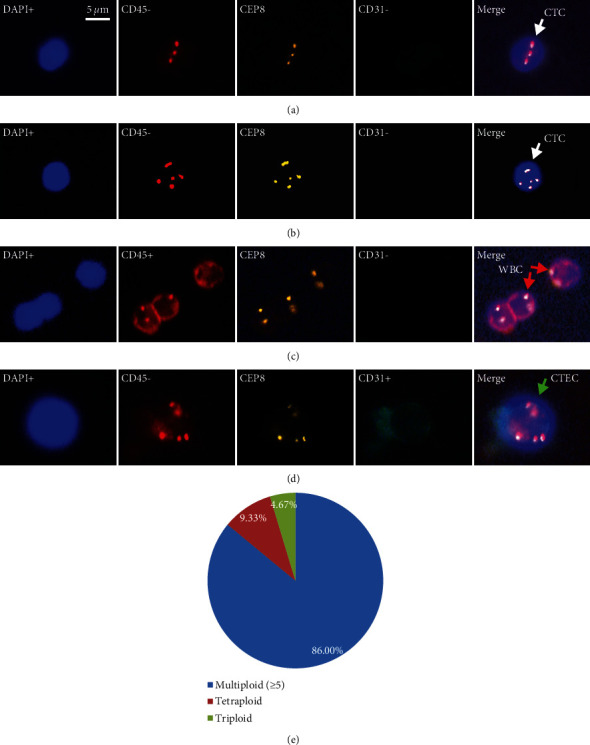
Images of CTCs and CTECs in CRC by subtraction enrichment and immunostaining-fluorescence in situ hybridization (SE-iFISH) under fluorescence microscope (×400). (a) DAPI+/CD45−/CEP8 = 3 (white arrow: triploid CTC); (b) DAPI+/CD45−/CEP8 > 5 (white arrow: multiploidy CTC); (c) DAPI+/CD45+/CEP8 = 2 (red arrow: WBC); (d) DAPI+/CD45−/CD31+/CEP8 > 5 (green arrow: CTEC); (e) distribution of CTCs according to ploidy. DAPI: blue, CD45: red, CEP8: orange, CD31: green.

A DAPI+/CD45−/CD31−/CEP8 > 2 was identified as CTC (Figures 1(a) and 1(b)), and count of CTCs ≥ 1 cells/7.5 mL was considered positive for peripheral blood CTCs. The interference by leukocytes (Figure 1(c)) and CTECs should be excluded using CD45+ and CD31+. CTECs were identified using the criterion DAPI+/CD45−/CD31+/CEP8 > 2 (Figure 1(d)).

### 2.5. CEA and CA19-9 Analysis

Venous blood (3 ml) was collected from every participant and centrifuged at 2100 × *g* for 10 min to get the corresponding serum. CEA and CA19-9 were detected by an automatic chemiluminescent immunoassay analyzer (cat. no. I2000SR; Abbott Laboratories). A CEA level >10.9 ng/mL was considered positive for CEA, and a level of CA19-9 > 37 U/ml was considered positive for this marker.

### 2.6. Statistical Analysis

SPSS20.0 (IBM Corp.), GraphPad Prism 6 (La Jolla, CA, USA), and *R* project were used for statistical analysis and graphical plots. Differences of categorical variables in distribution among groups were assessed with the Chi-square test or the Fisher exact test, as appropriate. Differences of continuous variables among groups were compared by Mann–Whitney *U* test or Kruskal–Wallis *H* test. The Pearson correlation method was used to analyze the correlation among variables. The diagnostic efficacy of each measure for CRC was evaluated using the receiver-operating characteristic (ROC) curve, and the diagnostic model was developed using binomial logistic regression, followed by the comparison of the area under each ROC curve (AUC). Results with *P* < 0.05 were considered statistically significant.

## 3. Results

### 3.1. CTCs and CTECs in CRC Patients

**Figure 2:**
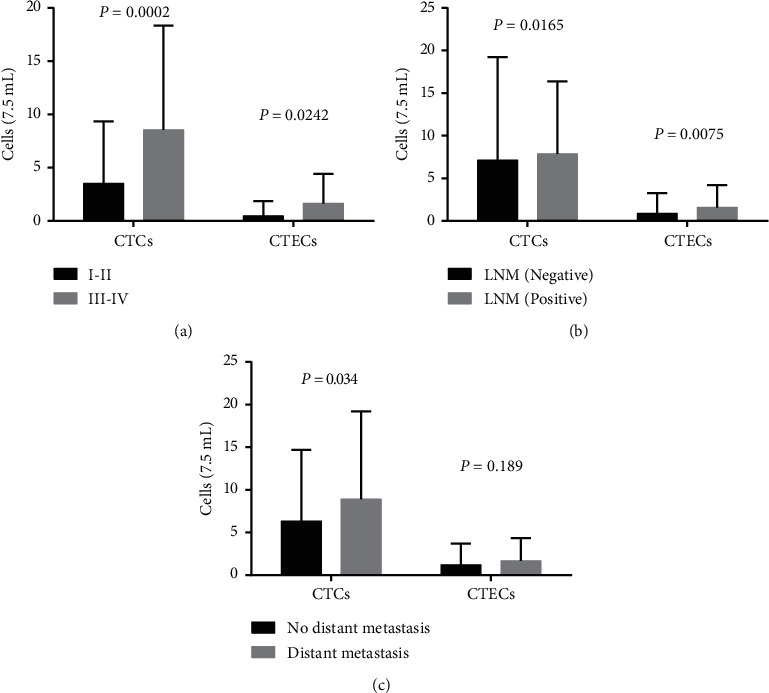
CTC counts in patients with CRC. (a) CTC and CTEC abundance between stage I-II and III-IV patients; (b) CTC and CTEC abundance according to lymph node metastasis; (c) CTC and CTEC abundance according to distant metastasis.

Table 1 shows the results of CTCs and CTECs according to clinical characteristics. The results of CTCs were positive in 101 CRC patients (87.83%, 101/115), and a total of 857 CTCs were detected. Thereinto, 4.67% CTCs (40/857) were triploid, 9.33% (80/857) were tetraploid, and 86% (737/857) were multiploid (CEP ≥ 5) (Figure 1(e)). Multiploid (CEP ≥ 5) CTCs were predominant over CTCs in CRC patients.  Figure 2 indicates CTC levels are significantly higher in advanced stage (Figure 2(a), *P*=0.0002), lymph node metastasis (LNM) (Figure 2(b), *P*=0.0165), and distant metastasis patients (Figure 2(c), *P*=0.034). Chi-square or Fisher exact test showed significant differences of CTC status in the clinical characteristics of patients including stage and LNM but no significant differences in gender, age, tumor location, differentiation, and distant metastasis (Table 1).

CTECs were detected in 45 CRC patients (39.13%, 45/115). Table 1 shows that significant differences in CTEC status in the clinical factors were consistent with CTCs' results.  Figure 2 shows CTECs' levels were significantly higher in advanced stage (Figure 2(a), *P*=0.0242) and LNM patients (Figure 2(b), *P*=0.0075) but not correlated with distant metastasis (Figure 2(c), *P*=0.189).

### 3.2. Comparison of CTCs, CTECs, CEA, and CA19-9 between Healthy Controls and CRC Patients

**Figure 3:**
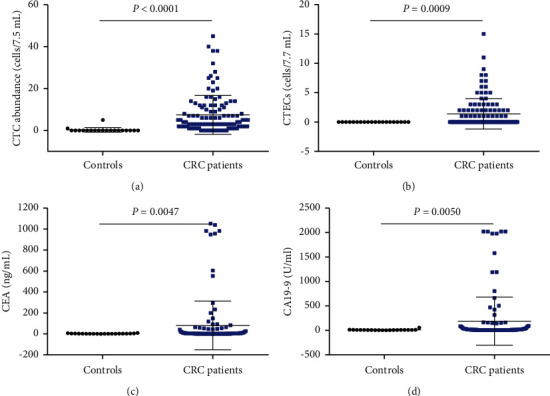
CTC (a), CTEC (b), CEA (c), and CA19-9 (d) levels between healthy controls and CRC patients.

Figure 3 shows the mean levels of CTCs (*P* < 0.0001), aneuploid CTECs (*P*=0.0009), CEA (*P*=0.0047), and CA19-9 (*P*=0.0050) are significantly higher in CRC patients than healthy controls.  Table 2 presents the performance of these four biomarkers to distinguish CRC patients from healthy controls based on clinical reference range. All four biomarkers presented excellent specificity, respectively, 0.900 of CTCs, 1 of CTECs, 1 of CEA, and 0.950 of CA19-9. Moreover, CTCs displayed the highest sensitivity of 0.878, while that of CTECs, CEA, and CA19-9 was undesirably 0.391, 0.287, and 0.261.

**Figure 4:**
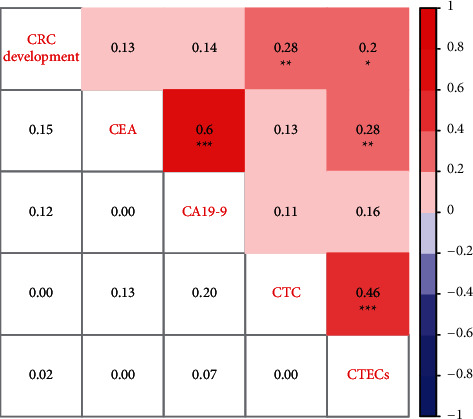
Heatmap showing the correlation between CTC, CTEC, CEA, CA19-9, and CRC development. The value in the grids of the upper triangle is the Pearson correlation coefficient (Pearson *r*), which is marked by colors. The value in grids of the lower triangle is *P* value of Pearson correlation.  ^*∗*^*P* < 0.05,  ^*∗*^ ^*∗*^*P* < 0.01, and  ^*∗*^ ^*∗*^ ^*∗*^*P* < 0.001.

We performed the Pearson correlation analysis between any two factors of CTCs, CTECs, CEA, CA19-9, and CRC development, and the results are shown in Figure 4. Only CTCs (*r* = 0.28, *P* < 0.01) and CTECs (*r* = 0.2, *P*=0.02) were significantly correlated with CRC development. Surprisingly, whether CEA (*r* = 0.13, *P*=0.15) or CA19-9 (*r* = 0.14, *P*=0.12) showed no striking correlation with CRC development. Besides, among four biomarkers, CA19-9 was only significantly correlated with CEA (*r* = 0.6, *P* < 0.001). CTC was only significantly associated with CTECs (*r* = 0.46, *P* < 0.001), unexpectedly not significantly associated with CEA or CA19-9. The above data indicated that CTCs were an independent biomarker of CRC development from CEA and CA19-9. The combination of multiple markers has the potential to widely improve CRC diagnosis efficiency.

### 3.3. CRC Diagnosis Performance by Combination of CTCs, CTECs, CEA, and CEA19-9

**Figure 5:**
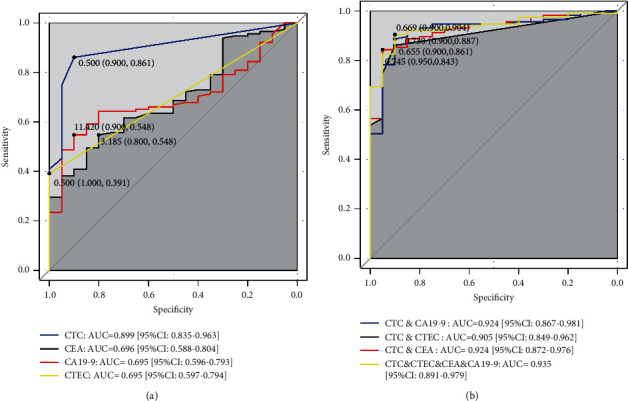
The ROC curve of biomarkers to distinguish CRC patients from healthy controls. (a) ROC curve of CTC, CTEC, CEA, and CA19-9, respectively; (b) ROC curve combination of CTC, CTEC, CEA, and CA19-9 by logistic regression.

We used the ROC curve to evaluate the efficiency of every biomarker to screen CRC patients from controls, and the results are shown in Figure 5(a). The CTC got the highest AUC score of 0.899, which possessed a high advantage compared with CTECs (AUC = 0.695), CEA (AUC = 0.696), and CA19-9 (AUC = 0.695). The optical cutoff value of CTCs and CTECs was both ≥1 cells/7.5 mL, while that of CEA and CA19-9 was, respectively, ≥3.185 ng/mL and ≥11.420 U/mL which is much lower than the clinical reference range.

Logistic regression models were constructed to combine multiple biomarkers to distinguish CRC patients. CTEC, CEA, and CA19-9 were singly combined with CTC by the logistic regression model, and the results indicated the addition of CEA or CA19-9 could improve the AUC score of CTC from 0.899 to 0.924 (Figure 5(b)). However, the addition of CTEC played a slight role in improving the AUC score from 0.899 to 0.905. The reason maybe CTEC was significantly correlated with CTC, while CEA and CA19-9 were independently on CTC (Figure 4). The combination of CTC, CTEC, CEA, and CA19-9 by logistic regression presented the best performance with 0.935 of AUC (Figure 5(b)).

## 4. Discussion

The incidence of CRC has been increasing annually, and metastasis and locally advanced stage in diagnosis are significant factors contributing to CRC-related mortality. Fortunately, early diagnosis and intervention can significantly reduce the mortality rate, and the 5-year survival rate of early postoperative patients can reach 90%–95% [20]. Patients with local invasion or distant metastases at initial diagnosis have a much lower 5-year survival rate (12%) [21]. Currently, the diagnosis of CRC is highly dependent on colonoscopy and pathological examination, which have significant limitations because of aggression. Although the acceptance of serum tumor biomarkers is high, the sensitivity of the commonly used CEA and CA19-9 is very low.

The migration of CTCs appears to be an early event in human carcinogenesis. Reportedly, CTCs have been detected in the blood of model animals when the tumor diameter was less than 1 mm [22]. In addition, it has been found that approximately 80% of metastatic tumor cells are derived from early disseminated cancer cells [23]. Reportedly, a meta-analysis of the prognostic significance of CTC count in CRC showed that the presence of CTCs in peripheral blood had a clear correlation with the overall survival and progression-free survival of patients [24]. This provides the theoretical basis for applying the CTC detection technique in the auxiliary diagnosis of CRC [25]. At present, CTC enrichment mainly depends on positive or negative enrichment methods. In recent years, it has been reported that tumor cells undergo EMT during entry into the blood circulation system, resulting in the downregulation of epithelial markers. Therefore, positive enrichment methods depending on epithelial marker often result in missed detection and false negatives.

SE-iFISH was a recently developed method for CTCs enrichment and detection without the above limitations. SE-iFISH could effectively remove leukocytes through the leukocyte marker CD45. Theoretically, chromosomal heteroploidy is regarded as the hallmark feature for identifying tumor cells by fluorescence in situ hybridization in this method [26]. Ge et al. used SE-iFISH to detect CTCs in peripheral blood derived from lung cancer and esophageal cancer with the detection rates of 92% and 87%, respectively [27]. The sensitivity and specificity of CTCs in pancreatic cancer patients were 88% and 90%, respectively [13]. Sheng et al. detected CTCs in 45 patients with breast cancer at a positive rate of 91% [28]. Overall, SE-iFISH has a higher detection rate for CTCs than the early FDA-approved CellSearch system. However, heteroploid CTECs interference was not distinguished by the above studies when detecting heteroploid CTCs.

In this study, we used an improved SE-iFISH, which distinguished heteroploid CTECs by anti-CD31 antibody improving the specificity of CTC recognition [19]. Then, 115 CRC patients and 20 healthy controls were enrolled, and heteroploid CTCs, CTECs, CEA, and CA19-9 were all tested in the blood. As the previous studies reported, we also found that the sensitivity of CEA and CA19-9 for diagnosing CRC was only 28.7% and 26.1%, respectively, while CTCs showed the highest positive rate of 87.8% in CRC patients, at the same time with good specificity of 90%. The detection rate was still high at 72.0% (18/25) for early-stage CRC (stage I and II). These rates are essential for the auxiliary diagnosis of early-stage CRC in patients. We also found not only a positive rate but also a mean level of CTCs was significantly higher in CRC patients with advanced stage and LNM, which was consistent with previous studies [29]. Interestingly, the similar phenomena appeared on CTECs of CRC patients, and CTECs' positive rate and mean level were also related with tumor's stage and LNM. Wang et al. reported that CTC-positive rate is positively associated with the level of serum CEA (*P*=0.001) in advanced CRC patients during chemotherapy course [30]. We found a significant correlation between CTC and CTEC but no significant correlation between CTC and CEA/CA19-9.

To improve the auxiliary diagnostic efficacy for CRC, we performed ROC curve of CTC, CTEC, CEA, and CA19-9, respectively. CTC obtained the highest AUC score of 0.899. Our results indicated that the sensitivity of CTCs was much higher than that of CEA, CA19-9, or CTECs. Combined with CTC, CTEC, CEA, and CA19-9 by logistic regression, the AUC score of the ROC curve was increased to 0.935. This result indicated CTCs were an ideal tumor biomarker of CRC. Combining CTC, CTEC with routine CEA, and CA19-9 was an optimal strategy for CRC screening and auxiliary diagnosis.

There are a few limitations in this study. Firstly, CTC is not a specific biomarker of CRC, which could be detected in various epithelial tumors, such as breast, esophageal, gastric cancers. So, there is a limitation of CTC used in CRC screening, and it is more appropriate for CRC auxiliary diagnosis. In addition, the sample size in this study was small, especially for patients at stage I and II. The logistic regression model needs a large of samples to optimize in future studies.

## 5. Conclusions

In summary, compared with the two commonly used tumor markers CEA and CA19-9, CTC based on SE-iFISH has a higher detection rate in CRC patients, with good specificity. Importantly, a logistic regression model was constructed overall considering CTC, CTEC, CEA, and CA19-9, which showed excellent efficacy in assisting the auxiliary diagnosis of CRC.

## Figures and Tables

**Table 1:** Clinicopathological characteristics of CRC patients and results of CTCs and CTECs.

Characteristics	Total (*N* = 115)	CTCs		*P* value	CTECs		*P* value
		Positive (*N* = 101)	Negative (*N* = 14)		Positive (*N* = 45)	Negative (*N* = 70)	
Gender				0.415			0.435
Male	69	62	7		29	40	
Female	46	39	7		16	30	
Age				0.754			0.102
≤60	62	55	7		20	42	
>60	53	46	7		25	28	
Location				0.333			0.572
Colon	60	51	9		22	38	
Rectum	55	50	5		23	32	
Differentiation				0.148			0.364
Poor	13	10	3		3	10	
Moderate/well	98	89	9		40	58	
Unknown	4	2	2		2	2	
Stage				0.006			0.010
I-II	25	18	7		4	21	
III-IV	90	83	7		41	49	
Distant metastasis				0.091			0.186
Yes	50	47	3		23	27	
No	65	54	11		22	43	
Lymph node metastasis				0.014			0.016
Yes	78	73	5		37	41	
No	28	21	7		6	22	
Unknown	9	7	2		2	7	

**Table 2:** The results of CTC, CTEC, CEA, and CA19-9 levels between CRC patients and controls.

Groups	CRC patients (*n* = 115)	Controls (*n* = 20)	*P* value	Sensitivity	Specificity
CTCs			<0.001	0.878	0.900
Positive	101 (87.8%)	2 (10.0%)			
Negative	14 (12.2%)	18 (90.0%)			
CTECs			<0.001	0.391	1
Positive	45 (39.1%)	0 (0%)			
Negative	70 (60.9%)	20 (100%)			
CEA			0.004	0.287	1
Positive	33 (28.7%)	0 (0%)			
Negative	82 (71.3%)	20 (100%)			
CA19-9			0.044	0.261	0.950
Positive	30 (26.1%)	1 (5.0%)			
Negative	85 (73.9%)	19 (95.0%)			

CTC and CTEC positive: ≥1 CTC/7.5 mL, CEA positive: >10.9 ng/mL, and CA19-9: >37 U/mL.

## Data Availability

The data used to support the findings of this study are included within the article.
